# Ischemic Heart Disease, Hematological Malignancies, and Infectious Diseases as Risk Factors for Cervical Cancer: A Study Based on Korean National Health Insurance Data

**DOI:** 10.3390/jcm14124282

**Published:** 2025-06-16

**Authors:** Heekyoung Song, Mirae Shin, Minji Seo, Yong-Wook Kim

**Affiliations:** 1 Department of Obstetrics and Gynecology, Incheon St. Mary’s Hospital, College of Medicine, The Catholic University of Korea, Seoul 06591, Republic of Korea; songdeng77@naver.com (H.S.); miraeshin1023@gmail.com (M.S.); 2 Department of Obstetrics and Gynecology, Nowon Eulji Medical Center, Eulji University, Seoul 01830, Republic of Korea

**Keywords:** cervical cancer, respiratory tuberculosis, ischemic heart disease, chronic rheumatic heart disease, chronic viral hepatitis, hematological malignancies

## Abstract

**Background/Objective:** Few studies have examined the direct relationship between cervical cancer and immune-related diseases. Therefore, this study aims to identify the association between cervical cancer and various underlying medical conditions using data from the Korean National Health Insurance System (NHIS). **Methods:** This retrospective cohort study was conducted using NHIS data from 2006 to 2022. A total of 1,344,628 women aged 19 years and older were included, of whom 68,275 were diagnosed with cervical cancer. Comorbidities were evaluated. The statistical analyses conducted included independent t-tests, chi-square tests, and multivariate logistic regression models to determine relative risks (RRs) and 95% confidence intervals (CIs). **Results:** The mean age of the patients with cervical cancer was significantly lower than that of the general population group. Body mass index and hypertension prevalence were higher in the cervical cancer group than in the general population group. Significant associations were observed between cervical cancer and respiratory tuberculosis (RR: 1.32, 95% CI: 1.27–1.38, *p* < 0.001), ischemic heart disease (RR: 1.72, 95% CI: 1.69–1.76, *p* < 0.001), chronic rheumatic heart disease (RR: 1.53, 95% CI: 1.44–1.64, *p* < 0.001), chronic viral hepatitis (RR: 1.33, 95% CI: 1.31–1.36, *p* < 0.001), and hematological malignancies (RR: 1.87, 95% CI: 1.67–2.09, *p* < 0.001). Conversely, cerebrovascular disease was associated with a reduced risk of cervical cancer (RR: 0.58, 95% CI: 0.57–0.60, *p* < 0.001). **Conclusion:** This study highlights the increased risk of cervical cancer in individuals with specific underlying diseases. These findings underscore the need for tailored screening and prevention strategies in high-risk populations.

## 1. Introduction

Cervical cancer is the fourth most common cancer among women worldwide and remains a leading cause of cancer-related mortality, particularly in developing countries [1]. In 2020, the age-standardized incidence rate of cervical cancer in South Korea per 100,000 individuals decreased to 3.7 from 4.4 per 100,000 in 2018 [2]. Although the incidence of cervical cancer has decreased worldwide, cervical cancer is still important to women’s health.

Human papillomavirus (HPV) infection is well established as the primary cause of cervical cancer, accounting for >90% of cases [3]. In particular, persistent infection with high-risk HPV represents the most significant risk factor for the development of high-grade cervical intraepithelial neoplasia [4]. Persistent HPV infection is associated with immune evasion mechanisms [5] and results from a multifactorial process influenced by immune response failure, the presence of high-risk HPV types, genetic instability of the lesion, microabrasions in the epithelium, and various host and environmental factors [6]. Human immunodeficiency virus (HIV) and HPV have a synergistic relationship, meaning that each virus can exacerbate the effects of the other [7]. Studies in mice have demonstrated that genetic abnormalities associated with Fanconi anemia can enhance the activity of oncogenic HPV [8]. Therefore, other viral infections, microbiome infections, or other immune statuses are related to cervical cancer occurrence due to HPV infection. However, few studies have examined the direct relationship between cervical cancer and immune-related diseases, such as other hepatitis or hematological diseases.

This study retrospectively analyzes the medical records of patients with cervical cancer using Korean national insurance databases to investigate the association between certain medical diseases and the development of cervical cancer. By exploring this association, we aimed to identify the potential risk factors that could be used to improve cervical cancer screening and prevention strategies in clinical practice.

## 2. Materials and Methods

### 2.1. Study Design and Population

**Figure 1:**
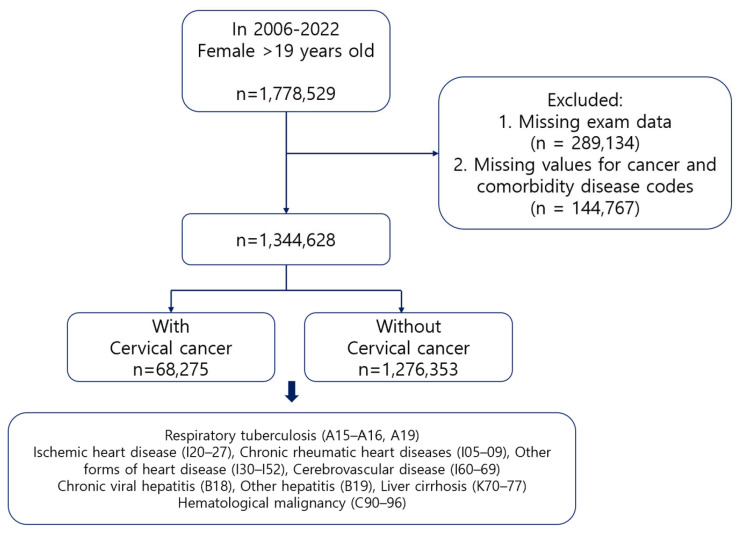
Flow chart of participant selection. Disease codes in this figure refer to the International Classification of Diseases, 10th Revision (ICD-10).

This retrospective analysis was based on data from the Korean National Health Insurance System (NHIS). The NHIS database includes sociodemographic data, laboratory results from health checkups, surgical codes, diagnostic codes for diseases, and causes of death. It also includes information on individual lifestyle factors such as cigarette smoking, alcohol consumption, and physical activity. From 1 January 2006 to 31 December 2022, women aged 19 years and older were included in this study, excluding those without information on health checkups or a diagnostic disease code. A total of 1,344,628 participants were included in this study; among them, 68,275 were diagnosed with cervical cancer and 1,276,353 had no history of cancer. A detailed flowchart is shown in Figure 1.

### 2.2. Data Collection

Comorbidities were recorded on the basis of the International Classification of Diseases, tenth revision codes for cervical cancer (C53), and the date of diagnosis. To reduce bias, cervical cancer stage 0 (C539.001), overlapping lesions of cervical cancer (C538), and small-cell carcinoma of the cervix (C539.008) were excluded. We evaluated the relative risk (RR) of underlying diseases in patients with cervical cancer. Hypertension (I10–I13, I15), diabetes mellitus (E10–E14), respiratory tuberculosis (A15–A16, A19), disease of the circulating system (ischemic heart disease [I20–27], chronic rheumatic heart diseases [I05–09], other forms of heart disease such as valve disease [I30–I52], and cerebrovascular disease [I60–69]), disease of the liver (chronic viral hepatitis (B18), other hepatitis (B19), and liver cirrhosis (K70–77)), and hematological malignancy (C90–96) were used to evaluate risk factors of cervical cancer. All underlying diseases were diagnosed before cervical cancer was diagnosed. Health checkup data, including age, body mass index (BMI), blood pressure (BP), hemoglobin, and total cholesterol levels were obtained from the national database.

### 2.3. Statistical Analysis

Continuous variables, including age, BMI, BP, hemoglobin, and total cholesterol levels were compared between patients with cervical cancer and controls using independent *t*-tests. For categorical variables, such as hypertension, diabetes mellitus, and smoking status, the chi-square test was used to assess differences in frequency between the groups.

A univariate analysis was performed to compare the incidence of cervical cancer based on the presence or absence of pre-existing conditions. RRs and 95% confidence intervals (CIs) were calculated for each variable. To control for potential confounding variables, a multivariate logistic regression analysis was conducted with BMI, hypertension, and diabetes mellitus as covariates, providing adjusted RR values. The statistical significance was set at *p* < 0.05, and the analyses were conducted SAS version 9.4 and SAS Viya (SAS Institute Inc., Cary, NC, USA).

### 2.4. Ethics Statements

This study was conducted in accordance with the ethical principles set forth in the Declaration of Helsinki and received approval from the Catholic Medical Center Institutional Review Board (approval number: OC23ZISI0058). Informed consent was not required because all patient data had been anonymized before analysis to protect patient confidentiality. Additionally, this study adheres to the STROBE (Strengthening the Reporting of Observational Studies in Epidemiology) guidelines to ensure methodological rigor and transparent reporting.

## 3. Results

### 3.1. Participant Characteristics

Table 1 summarizes the baseline characteristics of patients with and without cervical cancer. The mean age was significantly lower in the cervical cancer group than in the general population group (52.45 ± 13.55 years versus [vs.] 57.78 ± 13.05 years, *p* < 0.001). BMI and BP were significantly higher in the cervical cancer group than in the general population group (BMI: 23.49 ± 3.55 vs. 23.21 ± 3.51; systolic BP: 120.9 ± 16.28 mmHg vs. 119.3 ± 15.71 mmHg; diastolic BP: 74.95 ± 10.37 mmHg vs. 74.11 ± 10.22 mmHg; all *p* < 0.001). Additionally, the prevalence of hypertension was significantly higher in the cervical cancer group than in the general population group (41.62% vs. 31.35%; *p* < 0.001). However, diabetes mellitus and current smoking rates were lower in the cervical cancer group than in the general population group (diabetes mellitus: 24.95% vs. 41.68%, smoking rate: 9.46% vs. 10.86%; all *p* < 0.001).

Hemoglobin levels were marginally but statistically significantly lower in the cervical cancer group than in the general population group (12.84 ± 1.30 g/dL vs. 12.85 ± 1.26 g/dL; *p* = 0.0052). In contrast, total cholesterol levels were slightly higher in the cervical cancer group than in the general population group (197.5 ± 40.57 mg/dL and 195.6 ± 39.30 mg/dL, respectively; *p* = 0.0245).

### 3.2. Prevalence of Underlying Diseases in the Study Groups

A comparison of the prevalence of cervical cancer between individuals with and without underlying diseases revealed significant differences (Table 2). Conditions such as respiratory tuberculosis, ischemic heart disease, chronic rheumatic heart diseases, and other forms of heart disease, including valve disease, and chronic viral hepatitis, were more common in the cervical cancer group than in the general population group, suggesting a potential association with increased cancer risk. Notably, hematological malignancy was significantly more prevalent in the cervical cancer group than in the general population group, indicating a possible link between immunosuppression and cancer susceptibility. In contrast, cerebrovascular disease was less common in the cervical cancer group than in the general population group, underscoring a distinct risk profile.

### 3.3. RR of Cervical Cancer Based on Underlying Diseases

In the univariate analysis, individuals with a history of respiratory tuberculosis, ischemic heart disease, chronic rheumatic heart diseases, and other forms of heart disease, including valve disease, chronic viral hepatitis, or hematological malignancy, had a significantly higher incidence of cervical cancer than those without cervical cancer. In contrast, patients with cerebrovascular disease had a lower incidence of cervical cancer than those without. Among other diseases of the liver, no statistically significant difference in cervical cancer incidence was observed in patients with liver cirrhosis or other hepatitis (Table 3).

After adjusting for BMI, hypertension, and diabetes mellitus, a multivariate analysis was conducted. Respiratory tuberculosis was associated with a significantly increased risk of cervical cancer (RR: 1.32, 95% CI: 1.27–1.38, *p* < 0.001), demonstrating a higher incidence in the cervical cancer group than in the general population group. Among diseases of the circulating system, RRs for cervical cancer ranged from 1.53 (95% CI: 1.44–1.64) for chronic rheumatic heart diseases to 1.72 (95% CI: 1.69–1.76) for ischemic heart disease (all *p* < 0.001), whereas those for cerebrovascular disease showed a reduction (RR: 0.58, 95% CI: 0.57–0.60, *p* < 0.001). Notably, individuals with hematological malignancy were nearly 1.9 times more likely to be diagnosed with cervical cancer than the general population (RR: 1.87, 95% CI: 1.67–2.09, *p* < 0.001).

## 4. Discussion

Cervical cancer is associated with several well-established risk factors, including high-risk HPV infection [3], early sexual initiation with multiple sexual partners [9], smoking [10], and immunocompromised status [11]. This study is unique in that it uses large-scale Korean national data to explore the relationship between various the underlying conditions that may affect the immune system and the development of cervical cancer, thereby identifying novel risk factors. The findings indicate an increased risk of cervical cancer in patients with respiratory tuberculosis, ischemic heart disease, chronic rheumatic heart diseases, other forms of heart diseases, chronic viral hepatitis, and hematological malignancies.

With the identification of a necessary causative infectious agent [12] and the potential for managing populations with underlying diseases, cervical cancer is considered to be a highly preventable malignancy. Moreover, these results provide a valuable foundation for improving screening and management strategies for high-risk groups and may contribute to the World Health Organization’s Cervical Cancer Elimination Program [13].

In the present study, the incidence of cervical cancer was significantly increased in patients diagnosed with respiratory tuberculosis. Similarly, a Chinese retrospective study reported a significantly higher risk of cervical cancer in patients with tuberculosis (odds ratio [OR]: 3.49, 95% CI: 2.79–4.38) [14]. According to the national data of Lithuania, the risk of cervical cancer in patients with tuberculosis also increased, with an OR of 2.60 (CI: 1.93–3.42) [15]. Such findings may be attributed to the prolonged inflammatory response caused by tuberculosis, as well as the increased susceptibility to tuberculosis observed in individuals with HPV infection. Moreover, infection with *Mycobacterium tuberculosis* impairs dendritic cell function, which may weaken the host’s immune response to infectious agents and cervical cancer [16].

Among patients with disease of the liver, those diagnosed with only chronic viral hepatitis showed an increased incidence of cervical cancer. Previous studies have suggested that immune microenvironment alterations (e.g., infiltration of immunosuppressive cytokines), epigenetic changes (e.g., N6-methyladenosine modification), the dysregulation of molecular signaling pathways (including PI3K-Akt and Wnt), and serum biomarkers like the hepatitis B virus (HBV) X protein may contribute to the development of extrahepatic cancers associated with chronic HBV infection [17]. Consistent with our findings, a large hospital-based study conducted in Korea involving 2370 patients with cervical cancer reported a hepatitis B surface antigen (HBsAg) rate of 4.98% and identified a significant association between HBV infection and cervical cancer risk (adjusted OR = 1.49) [18]. Interestingly, a lower risk was observed in patients older than 60 years of age with HBV infection in another study (adjusted OR = 0.73) [19]. Since our study population primarily consisted of individuals younger than 60 years of age (mean age: 52.45 years), our results align with those of previous research, indicating an increased risk of cervical cancer in the chronic viral hepatitis group.

In our analysis, we were unable to separately identify diagnostic codes for HBV and hepatitis C virus (HCV), and therefore investigated chronic viral hepatitis caused by HBV and HCV collectively. Fewer studies have explored the association between chronic hepatitis C and cervical cancer than the association between chronic hepatitis B and cervical cancer. Luo et al. also examined the relationship between HCV-related hepatitis and cervical cancer; however, no statistically significant difference was found, likely due to the small size of the control group [20]. Using Brazilian prospective cohort data, HBV or HCV in the HIV-infected population was found to be highly associated with HPV or HIV infections [21]. However, only 103 individuals were enrolled, which was too small to determine the OR. Unlike chronic viral hepatitis, other types of hepatitis did not show an increased risk of cervical cancer. Although liver cirrhosis demonstrated statistical significance, the adjust RR was only 1.07, indicating that the increase in risk is not clearly evident. Therefore, further research is needed on chronic infections that may exert long-term immunological effects, and future studies should investigate the risk differences according to viral subtypes.

Here, patients with ischemic heart disease demonstrated a higher incidence of cervical cancer than those without. This finding aligns with results of previous reports suggesting that individuals with cardiovascular conditions may exhibit a higher prevalence of HPV infection than those without [22]. While a limited number of studies have explored this association, most of the existing literature has instead focused on the impact of HPV infection or cervical cancer on thrombotic events, which differs from the scope of the present analysis. For instance, HPV impairs vascular endothelial function and induces systemic inflammation [23], as well as disrupts host lipid metabolism, thereby contributing to the progression of atherosclerosis and ischemic heart disease [24]. Our findings, however, suggest that ischemic heart disease itself may predispose individuals to HPV infection and, consequently, increase the risk of cervical cancer.

Conversely, among diseases of the circulatory system, only patients diagnosed with cerebrovascular disease were found to have a decreased risk of cervical cancer. In a previous study of German cancer survivors following stroke, the incidence of gynecological cancers showed a slight increase after 10 years; however, the difference was not statistically significant (OR: 1.08, 95% CI: 0.88–1.33, *p* = 0.457) [25]. In our cohort, ischemic heart disease and cerebrovascular disease were identified in 18,300 (6.39%) and 7088 (2.65%) patients, respectively. Despite its lower prevalence, disability resulting from stroke is significantly more common. Among Korean women, disability due to cerebrovascular disease accounts for 9.5%, compared to only 0.16% for cardiac causes [26]. This disparity suggests that cervical cancer may be underdiagnosed in stroke patients due to reduced participation in screening programs. Furthermore, stroke-related impairments may limit not only access to screening, but also sexual activity, potentially reducing exposure to human papillomavirus (HPV)—the primary etiological agent in cervical cancer development [27]. Further prospective studies are warranted to elucidate the differential impact of cardiovascular and cerebrovascular conditions on cervical cancer development.

Building upon these observations, the present study additionally identifies a significant association between cervical cancer and several cardiac conditions, particularly chronic rheumatic heart disease and other heart diseases, including valvular disorders. Chronic rheumatic heart disease, which results from an autoimmune response to group A streptococcal infection [28], may reflect underlying immune dysfunction—similar to that observed in chronic viral hepatitis or tuberculosis—that could increase susceptibility to HPV infection and, ultimately, cervical cancer. However, the increased risk observed in patients with other heart diseases, such as valvular disorders, remains unclear and merits further investigation. These findings demonstrate the importance of close surveillance in individuals with pre-existing cardiac conditions, including chronic ischemic heart disease, due to their heightened risk of developing cervical cancer.

The adjusted RR for hematological malignancies was 1.87, which was higher than that for other conditions. This finding is consistent with the results of earlier studies that indicate that the incidence of cervical cancer following hematopoietic stem cell transplantation (HCT) was at least 13 times higher than that in the general population [29]. Immunosuppression after HCT increases the incidence of HPV-associated cervical dysplasia [30], and international blood and marrow transplant societies recommend that patients with hematological malignancies who have undergone HCT undergo annual HPV and Papanicolaou testing [29]. As this study included patients diagnosed with hematological malignancies regardless of HCT, it may have included a large number of patients receiving follow-up after HCT, which may have resulted in a higher RR of cervical cancer than that for other comorbidities.

This study has some limitations. It is based on data from the NHIS, which provides information from general health checkups, including diagnostic codes and basic laboratory findings. However, it did not distinguish between chronic viral infections caused by HBV and HCV, nor did it include detailed laboratory markers such as HBV antigen levels or the presence of seroconversion. An et al. reported that HBsAg seropositivity increased the risk of cervical cancer [18]; however, we did not make this conclusion. Moreover, diagnostic information on HIV was not provided to general researchers due to ethical issues; therefore, we could not evaluate HIV infection and cervical cancer diagnosis, even though several reports have reported increased rates of HPV persistence in individuals living with HIV and those with compromised immune systems [11,31]. One study found that HPV infection was associated with a 2.5-fold increased risk of HIV infection among women. While causality has not been proven, overlapping vaginal cytokine profiles suggest a potential link between HPV-induced inflammation and increased susceptibility to HIV infection [32]. Furthermore, due to limitations of the current NHIS dataset, we were unable to assess HPV vaccination status [33] and cancer staging, both of which are critical for cervical cancer prevention or clinical interpretation. The inability to analyze these important factors represents a key limitation of our study. Additionally, as this study was based on NHIS data, it was not possible to establish clear causal relationships between each diagnosis and the increased incidence of cervical cancer. The results may also have been influenced by confounding variables and missing data. Therefore, future studies should include mechanistic investigations and long-term follow-up to better elucidate the association between cervical cancer development and underlying medical conditions.

Despite these limitations, the novelty of this study lies in its use of a large national database, which facilitated the analysis of the association between specific medical conditions and the development of cervical cancer. As a multivariate analysis was performed, the results may be more robust than those of previous findings. This is the most substantial strength of this study. Furthermore, ischemic heart disease, chronic rheumatic heart diseases, and hematological malignancy, which have been evaluated less frequently in previous analyses, were found to be significant risk factors for cervical cancer. Our results provide significant evidence for cancer prevention and management strategies. Although our study employed a retrospective design, the long-term follow-up period and large sample size enhance the reliability of our findings.

## 5. Conclusions

Individuals with specific underlying diseases, particularly respiratory tuberculosis, ischemic heart disease, chronic rheumatic heart disease, chronic viral hepatitis, and hematological malignancies, have an increased risk of cervical cancer. These findings underscore the need for tailored screening and prevention strategies in high-risk populations.

## Figures and Tables

**Table 1:** Participants’ baseline characteristics.

	Cervical Cancer (Mean ± SD)	General Population (Mean ± SD)	*p*-Value
Age (years)	52.45 (13.55)	57.78 (13.05)	<0.001
BMI (kg/m^2^)	23.49 (3.55)	23.21 (3.51)	<0.001
Systolic BP (mmHg)	120.9 (16.28)	119.3 (15.71)	<0.001
Diastolic BP (mmHg)	74.95 (10.37)	74.11 (10.22)	<0.001
Hypertension ^a^			<0.001
Yes	531,260 (41.62%)	21,407 (31.35%)	
No	745,093 (58.38%)	46,868 (68.65%)	
Diabetes mellitus ^b^			<0.001
Yes	17,034 (24.95%)	531,944 (41.68%)	
No	51,241 (75.05%)	744,409 (58.32%)	
Smoking ^c^			<0.001
Yes	120,697 (9.46%)	7414 (10.86%)	
No	1,155,656 (90.54%)	60,861 (89.14%)	
Hemoglobin level (g/dL)	12.84 (1.30)	12.85 (1.26)	0.0052
Total cholesterol level (mg/dL)	197.5 (40.57)	195.6 (39.30)	0.0245

^a^ Individuals were classified as having hypertension if their diagnostic codes included hypertension (I10–I13, I15). ^b^ Individuals were classified as having diabetes mellitus if their diagnostic codes included diabetes mellitus (E10–E14). ^c^ Smoking status was categorized as “yes” for past and current smokers. BMI, body mass index; BP, blood pressure; SD, standard deviation.

**Table 2:** Prevalence of underlying diseases in cervical cancer and general population groups.

Underlying Disease ^a^	Cervical Cancer, n (%)	General Population, n (%)
Respiratory tuberculosis (A15–16, A19)	2549 (6.77%)	54,939 (5.36%)
Disease of the circulating system		
- Ischemic heart disease (I20–27)	18,300 (6.39%)	39,188 (5.30%)
- Chronic rheumatic heart diseases (I05-09)	960 (6.57%)	56,528 (5.39%)
- Other forms of heart disease (I30–I52)	18,461 (6.51%)	39,027 (5.26%)
- Cerebrovascular disease (I60–69)	7088 (2.65%)	61,187 (5.68%)
Disease of the liver		
- Chronic viral hepatitis (B18)	9321 (6.77%)	48,167 (5.43%)
- Other hepatitis (B19)	1063 (5.62%)	56,425 (5.61%)
- Liver cirrhosis (K70–77)	5219 (5.66%)	52,269 (5.60%)
Hematological malignancy (C90–96)	57,179 (5.60)	309 (8.86)

^a^ All disease codes in this table refer to the ICD-10.

**Table 3:** Relative risk of cervical cancer based on underlying diseases.

Underlying Disease ^a^	Univariate RR (95% CI)	*p*-Value	Multivariate RR (95% CI) ^b^	*p*-Value
Respiratory tuberculosis (A15–16, A19)	1.22 (1.17–1.26)	<0.001	1.32 (1.27–1.38)	<0.001
Disease of the circulating system				
- Ischemic heart disease (I20–27)	1.21 (1.18–1.22)	<0.001	1.72 (1.69–1.76)	<0.001
- Chronic rheumatic heart diseases (I05–09)	1.17 (1.10–1.25)	<0.001	1.53 (1.44–1.64)	<0.001
- Other forms of heart disease (I30–I52)	1.24 (1.22–1.26)	<0.001	1.60 (1.57–1.63)	<0.001
- Cerebrovascular disease (I60–69)	0.47 (0.46–0.48)	<0.001	0.58 (0.57–0.60)	<0.001
Disease of the liver				
- Chronic viral hepatitis (B18)	1.25 (1.22–1.28)	<0.001	1.33 (1.31–1.36)	<0.001
- Other hepatitis (B19)	1.00 (0.94–1.06)	0.961	1.10 (1.03–1.17)	0.0025
- Liver cirrhosis (K70–77)	0.99 (0.96–1.02)	0.46	1.07 (1.04–1.10)	<0.001
Hematological malignancy (C90–96)	1.58 (1.42–1.76)	<0.001	1.87 (1.67–2.09)	<0.001

^a^ All disease codes in this table refer to the ICD-10. ^b^ A multivariate logistic regression analysis was conducted with body mass index, hypertension, and diabetes mellitus as covariates, providing adjusted RR values. CI, confidence interval; RR, relative risk.

## Data Availability

Individual participant data that underlie the results reported in this article, after de-identification, are available upon reasonable request from the corresponding author.

## Abbreviations

The following abbreviations are used in this manuscript:

HPV	human papillomavirus
HIV	human immunodeficiency virus
NHIS	National Health Insurance
CIs	confidence intervals
vs.	versus
RR	relative risk
CI	confidence interval
OR	odds ratio
HBV	hepatitis B virus
HBsAg	hepatitis B surface antigen
HCV	hepatitis C virus
HCT	hematopoietic stem cell transplantation
BMI	body mass index
BP	blood pressure

## References

[1] Sung H., Ferlay J., Siegel R.L., Laversanne M., Soerjomataram I., Jemal A., Bray F. (2021). Global cancer statistics 2020: GLOBOCAN estimates of incidence and mortality worldwide for 36 cancers in 185 countries. CA Cancer J. Clin..

[2] Kang M.J., Jung K.W., Bang S.H., Choi S.H., Park E.H., Yun E.H., Kim H.J., Kong H.J., Im J.S., Seo H.G. (2023). Community of Population-Based Regional Cancer Registries. Cancer statistics in Korea: Incidence, mortality, survival, and prevalence in 2020. Cancer Res. Treat..

[3] Georgieva S., Iordanov V., Sergieva S. (2009). Nature of cervical cancer and other HPV—Associated cancers. J. BUON.

[4] Rositch A.F., Koshiol J., Hudgens M.G., Razzaghi H., Backes D.M., Pimenta J.M., Franco E.L., Poole C., Smith J.S. (2013). Patterns of persistent genital human papillomavirus infection among women worldwide: A literature review and meta-analysis. Int. J. Cancer.

[5] Choi Y.J., Park J.S. (2016). Clinical significance of human papillomavirus genotyping. J. Gynecol. Oncol..

[6] Stanley M. (2010). Pathology and epidemiology of HPV infection in females. Gynecol. Oncol..

[7] Looker K.J., Rönn M.M., Brock P.M., Brisson M., Drolet M., Mayaud P., Boily M.C. (2018). Evidence of synergistic relationships between HIV and human papillomavirus (HPV): Systematic reviews and meta-analyses of longitudinal studies of HPV acquisition and clearance by HIV status, and of HIV acquisition by HPV status. J. Int. AIDS Soc..

[8] Park S., Park J.W., Pitot H.C., Lambert P.F. (2016). Loss of dependence on continued expression of the human papillomavirus 16 E7 oncogene in cervical cancers and precancerous lesions arising in fanconi anemia pathway-deficient mice. mBio.

[9] Winer R.L., Lee S.K., Hughes J.P., Adam D.E., Kiviat N.B., Koutsky L.A. (2003). Genital human papillomavirus infection: Incidence and risk factors in a cohort of female university students. Am. J. Epidemiol..

[10] Roura E., Castellsagué X., Pawlita M., Travier N., Waterboer T., Margall N., Bosch F.X., de Sanjosé S., Dillner J., Gram I.T. (2014). Smoking as a major risk factor for cervical cancer and pre-cancer: Results from the EPIC cohort. Int. J. Cancer.

[11] Palefsky J.M., Holly E.A. (2003). Immunosuppression and co-infection with HIV. J. Natl. Cancer Inst. Monogr..

[12] Castle P.E., Einstein M.H., Sahasrabuddhe V.V. (2021). Cervical cancer prevention and control in women living with human immunodeficiency virus. CA Cancer J. Clin..

[13] World Health Organization Cervical Cancer Elimination Initiative. https://www.who.int/initiatives/cervical-cancer-elimination-initiative.

[14] Chen G.L., Guo L., Yang S., Ji D.M. (2021). Cancer risk in tuberculosis patients in a high endemic area. BMC Cancer.

[15] Everatt R., Kuzmickiene I., Davidaviciene E., Cicenas S. (2017). Non-pulmonary cancer risk following tuberculosis: A nationwide retrospective cohort study in Lithuania. Infect. Agent Cancer.

[16] Manickam A., Sivanandham M. (2011). Mycobacterium bovis BCG and purified protein derivative-induced reduction in the CD80 expression and the antigen up-take function of dendritic cells from patients with cervical cancer. Eur. J. Obstet. Gynecol. Reprod. Biol..

[17] Min Y., Wei X., Xia X., Wei Z., Li R., Jin J., Liu Z., Hu X., Peng X. (2023). Hepatitis B virus infection: An insight into the clinical connection and molecular interaction between hepatitis B virus and host extrahepatic cancer risk. Front. Immunol..

[18] An J., Kim J.W., Shim J.H., Han S., Yu C.S., Choe J., Lee D., Kim K.M., Lim Y.S., Chung Y.H. (2018). Chronic hepatitis B infection and non-hepatocellular cancers: A hospital registry-based, case-control study. PLoS ONE.

[19] Lu T., Yang Q., Li M., Zhang J., Zou J., Huang L., Lin J., Jin H., He J. (2018). HBV infection and extra-hepatic cancers in adolescents and 20s: A retrospective study in China. Cancer Epidemiol..

[20] Luo C., Yu S., Zhang J., Wu X., Dou Z., Li Z., Yang E., Zhang L. (2022). Hepatitis B or C viral infection and the risk of cervical cancer. Infect. Agent Cancer.

[21] Bomfim-Hyppólito S., Eleuterio J., Nunes G.C., Bomfim-Hyppólito E., Franco E.S., Neto R.J.P. (2013). HIV or human papillomavirus co-infection among Brazilian individuals infected with hepatitis B and/or hepatitis C. Int. J. Gynecol. Obstet..

[22] Cheong H.S., Chang Y., Kim Y., Kwon M.J., Cho Y., Kim B., Joo E.J., Bae Y.H., Kim C., Ryu S. (2024). Human papillomavirus infection and cardiovascular mortality: A cohort study. Eur. Heart J..

[23] Mazibrada J., Rittà M., Mondini M., De Andrea M., Azzimonti B., Borgogna C., Ciotti M., Orlando A., Surico N., Chiusa L. (2008). Interaction between inflammation and angiogenesis during different stages of cervical carcinogenesis. Gynecol. Oncol..

[24] Bravo I.G., Crusius K., Alonso A. (2005). The E5 protein of the human papillomavirus type 16 modulates composition and dynamics of membrane lipids in keratinocytes. Arch. Virol..

[25] Jacob L., Kostev K. (2019). Cancer risk in stroke survivors followed for up to 10 years in general practices in Germany. J. Cancer Res. Clin. Oncol..

[26] Kim M., Jung W., Kim S.Y., Park J.H., Shin D.W. (2023). The Korea National Disability Registration System. Epidemiol. Health.

[27] Robledo-Resina I.d.M., Romero-Morales C., Martín-Casas P., Villafañe J.H., Abuín-Porras V. (2024). Relationship between Female Sexual Dysfunction and Trunk Stability Post-Stroke: A Cross-Sectional Study. Medicina.

[28] Marijon E., Mirabel M., Celermajer D.S., Jouven X. (2012). Rheumatic heart disease. Lancet.

[29] Inamoto Y., Shah N.N., Savani B.N., Shaw B.E., Abraham A.A., Ahmed I.A., Akpek G., Atsuta Y., Baker K.S., Basak G.W. (2015). Secondary solid cancer screening following hematopoietic cell transplantation. Bone Marrow Transplant..

[30] Savani B.N., Stratton P., Shenoy A., Kozanas E., Goodman S., Barrett A.J. (2008). Increased risk of cervical dysplasia in long-term survivors of allogeneic stem cell transplantation—Implications for screening and HPV vaccination. Biol. Blood Marrow Transplant..

[31] McNally G.A. (2019). HIV and cancer: An overview of AIDS-defining and non-AIDS-defining cancers in patients with HIV. Clin. J. Oncol. Nurs..

[32] Liebenberg L.J.P., McKinnon L.R., Yende-Zuma N., Garrett N., Baxter C., Kharsany A.B.M., Archary D., Rositch A., Samsunder N., Mansoor L.E. (2019). HPV infection and the genital cytokine milieu in women at high risk of HIV acquisition. Nat. Commun..

[33] Lei J., Ploner A., Elfström K.M., Wang J., Roth A., Fang F., Dillner J., Sparén P. (2020). HPV Vaccination and the Risk of Invasive Cervical Cancer. N. Engl. J. Med..

